# Chemokines Referee Inflammation within the Central Nervous System during Infection and Disease

**DOI:** 10.1155/2014/806741

**Published:** 2014-09-30

**Authors:** Douglas M. Durrant, Jessica L. Williams, Brian P. Daniels, Robyn S. Klein

**Affiliations:** ^1^Department of Internal Medicine, Washington University School of Medicine, Campus Box 8051, 660 S. Euclid Avenue, St. Louis, MO 63110, USA; ^2^Department of Anatomy and Neurobiology, Washington University School of Medicine, Campus Box 8051, 660 S. Euclid Avenue, St. Louis, MO 63110, USA; ^3^Department of Pathology and Immunology, Washington University School of Medicine, Campus Box 8051, 660 S. Euclid Avenue, St. Louis, MO 63110, USA

## Abstract

The discovery that chemokines and their receptors are expressed by a variety of cell types within the normal adult central nervous system (CNS) has led to an expansion of their repertoire as molecular interfaces between the immune and nervous systems. Thus, CNS chemokines are now divided into those molecules that regulate inflammatory cell migration into the CNS and those that initiate CNS repair from inflammation-mediated tissue damage. Work in our laboratory throughout the past decade has sought to elucidate how chemokines coordinate leukocyte entry and interactions at CNS endothelial barriers, under both homeostatic and inflammatory conditions, and how they promote repair within the CNS parenchyma. These studies have identified several chemokines, including CXCL12 and CXCL10, as critical regulators of leukocyte migration from perivascular locations. CXCL12 additionally plays an essential role in promoting remyelination of injured white matter. In both scenarios we have shown that chemokines serve as molecular links between inflammatory mediators and other effector molecules involved in neuroprotective processes.

## 1. Introduction

Chemokines are small, secreted proteins originally shown to promote the migration of leukocytes both during immune surveillance and in response to inflammation. Chemokine have been classified into CXC, CC, C, or CX_3_C subfamilies, according to the positions of conserved cysteine residues at their N-termini, and promiscuously bind receptor members of the G protein-coupled receptor superfamily [1]. Each chemokine or its receptor is named based on their subfamily designation with “L” indicating ligand and “R” indicating receptor plus a number, which corresponds to the same numbers used in the corresponding gene nomenclature. Binding of chemokines to their receptors generally results in calcium mobilization and cytoskeletal rearrangements required for cell motility in response to a signal of increasing chemokine concentration [2, 3]. Their initial discovery by immunologists led to an explosion of studies demonstrating their far-reaching roles in all aspects of immune function from immune surveillance and leukocyte interactions within lymph nodes to orchestration of innate and adaptive immune responses against invading pathogens.

The identification of two chemokine receptors, CCR5 and CXCR4, as critical coreceptors for the entry of HIV-1 into CD4^+^ T cells [4–6] heralded an intense period of chemokine research leading to the detection of these molecules on multiple cell types within the CNS in both homeostatic and inflammatory conditions. CXCR4, in particular, is expressed by neural progenitors, mature neurons, and endothelial cells in the normal adult CNS [7–10]. CXCR4 is the most highly conserved chemokine receptor throughout evolution [11, 12] with functional origins in the development of multiple organ systems, including the immune and central nervous systems (CNS) [13]. Its primary ligand, CXCL12, is also expressed by neurons and epithelium [14]. CXCL12 is the second most highly conserved chemokine ligand; CXCL14, another CNS chemokine, is the first and a natural inhibitor of CXCL12-CXCR4 interactions [15]. An additional receptor for CXCL12, CXCR7, functions to scavenge the chemokine and heterodimerize with CXCR4 in order to regulate CXCR4 signaling [16–18]. The identification of CXCL12/CXCR4/CXCR7 as primordial is consistent with their critical roles in the development of multiple organ systems and in their physiologic, pleiotropic functions in both immune and nervous systems. CXCL12 was first identified for its role in the homing of hematopoietic progenitor cells to the bone marrow and later as a facilitator of lymphocyte entry into lymph nodes during immune surveillance [19–22], the latter due to it expression along the lumenal surfaces of high endothelial venules. The detection of high levels of CXCL12 expressed by CNS endothelium was curious, as this site is normally regarded for its stringent regulation of lymphocyte entry. However, our laboratory soon discovered that the polarity of CXCL12 expression in the CNS was reversed compared to its expression within lymphoid tissues and ultimately established CXCL12 and its scavenger receptor, CXCR7, as critical molecular components of immune privilege and as orchestrators of efficacious antiviral responses, promoting requisite interactions between leukocytes that localize to perivascular spaces [23–27]. These interactions are critical for limiting the entry of immune cells into the CNS parenchyma to those that may swiftly eliminate pathogen and preserve CNS function.

Studies in chemokine neurobiology also shifted our understanding of the role of proinflammatory chemokine expression during neurologic diseases. Early studies had cast these molecules as immunopathologic targets whose inactivation would ameliorate disease by limiting inflammation. Investigations using both infectious and autoimmune models of neuroinflammation have since demonstrated that chemokine expression within the CNS may be neuroprotective. For example, our laboratory demonstrated that neurons infected with the neurotropic flavivirus West Nile virus (WNV) express T lymphocyte chemoattractants, such as CXCL10, to promote adaptive immune responses within the CNS to clear virus [28, 29]. These studies were also the first to delineate differences in innate immune responses of the hindbrain and forebrain as WNV-infected cerebellar granule cell neurons exhibit brisk upregulation of CXCL10 expression compared with infected cortical neurons, which protects the cerebellum from extensive viral infection. We also demonstrated that while CXCL12 does not mediate leukocyte recruitment into the CNS, increased parenchymal expression of this chemokine during neuroinflammatory states promotes CNS repair, harkening back to its developmental roles in neural progenitor cell proliferation and differentiation [30–32]. In this review, we discuss studies from our laboratory that have established fundamental and functional roles for chemokines at CNS endothelial barriers and in the protection and repair of the CNS parenchyma.

## 2. Chemokines Function as Molecular Components of Immune Privilege at the Blood-Brain Barrier

Among the most influential sites of regulation for chemokine signaling in the CNS is the blood-brain barrier (BBB). The BBB is a complex physiological interface between the hematogenous circulation and parenchymal CNS tissues, composed of brain microvascular endothelial cells (BMECs) joined by a network of tight junctions (TJs) and adherens junctions (AJs) [33]. BMECs are ensheathed by pericytes and the endfeet processes of adjacent astrocytes, both of which contribute to TJ and AJ formation and enhance the barrier properties of BMECs [34]. Chemokine signaling at the BBB occurs in all neurovascular cell types, though their actions in endothelial cells and astrocytes are most well understood. Immune cells that infiltrate the CNS via the circulation first enter a space between vascular endothelia and glial endfeet known as the perivascular space, in which they are subject to the regulatory actions of chemokines and other CNS immune factors.

In BMECs, the expression and localized display of CXCL12 is known to be a major regulator of CNS immune privilege. Under homeostatic conditions, CXCL12 is expressed exclusively along basolateral/ablumenal surfaces of BMECs [24, 25]. Upon extravasation into the perivascular space, CXCR4^+^ leukocytes are captured by endothelial CXCL12, restricting them to the perivascular space and preventing their parenchymal infiltration [24, 25]. This process is responsible for much of the “perivascular cuffing” that occurs during inflammatory episodes of the CNS, as large numbers of infiltrating leukocytes accumulate on the basolateral surfaces of vascular endothelium. This restriction of leukocyte access to the CNS parenchyma is essential to limit immunopathology and bystander injury of neurons, which have limited capacity for repair in comparison to peripheral tissues [35, 36].

Due to this essential function, disease states that alter CXCL12 expression and localization at the BBB can lead to uncontrolled neuroinflammation, resulting in axonal injury, demyelination, and neuronal death. In the CNS autoimmune disease multiple sclerosis (MS) and its mouse model experimental autoimmune encephalomyelitis (EAE), myelin-specific T lymphocytes invade the CNS parenchyma, forming lesions characterized by demyelinated and injured axons. In both MS and EAE, the homeostatic localization of CXCL12 is disturbed, with loss of polarized expression along basolateral surfaces of BMECs. Loss of CXCL12 polarity results in impaired perivascular capture of infiltrating leukocytes, with subsequent accumulations of CXCR4^+^ cells in parenchymal tissues leading to progressive neuropathology. In mouse studies, blockade of CXCL12-CXCR4 interactions at the BBB via administration of the CXCR4 antagonist AMD3100 resulted in enhanced clinical disease severity during EAE, associated with enhanced parenchymal immune infiltrates, increased microglial activation, and increased CNS expression of Th1 inflammatory mediators [25]. In studies of tissue from MS patients, uninflamed vessels exhibited normal basolateral vascular expression of CXCL12, while inflammatory lesions contained vessels with CXCL12 expression that was redistributed to vessel lumena. Of note, the extent of pathological CXCL12 expression within MS lesions positively correlated with levels of neuroinflammation and demyelination [24]. Together, these studies established a critical role for CXCL12 at the BBB in the orchestration of leukocyte trafficking during CNS autoimmunity.

## 3. Mediators of CXCL12 Signaling during CNS Autoimmunity

Given the central role of CXCL12 at the BBB during homeostasis and neuroinflammation, understanding the mechanisms by which CXCL12 is regulated at this site is crucial in the development of targeted therapeutics. Endothelial CXCL12 is subject to regulation by a number of factors, including reciprocal regulation by infiltrating immune cells. In particular, mouse studies have established that expression of the inflammatory cytokine IL-1*β* contributes to loss of CXCL12 polarity in vascular endothelium during the induction of EAE, facilitating later CNS parenchymal invasion by autoreactive leukocytes [37]. During EAE, infiltrating CD4^+^, CD8^+^, and *γδ* T cells are sources of IL-1*β* within the CNS. IL1R^−/−^ mice are resistant to EAE, and, in contrast to WT controls, exhibit intact CXCL12 polarity at the BBB following myelin oligodendrocyte glycoprotein- (MOG-) immunization. Administration of recombinant IL-1*β* also potently induced the relocalization of CXCL12 to vessel lumena in spinal cords of naïve WT mice. Bone marrow chimera experiments revealed that IL1R signaling on both leukocytes and resident CNS cells contributed to changes in CXCL12 polarity at the BBB during EAE, suggesting that IL1R signaling is a key systemic regulator of CXCL12 within the inflamed CNS.

While leukocyte-derived factors play a role in regulating CXCL12 at the BBB, endothelial cells, themselves, express key regulators of CXCL12 expression and polarity that contribute to autoimmune pathogenesis. A key CXCL12 regulatory protein is the atypical chemokine receptor CXCR7/ACKR3 (CXCR7). CXCR7 has key regulatory functions in the CNS during development and homeostasis, regulating the migration of leukocytes, neural precursor cells, and immature neurons via signaling pathways distinct from those of CXCR4 [38, 39]. BMECs are among the primary expressers of CXCR7 within the CNS [23]. Endothelial CXCR7 serves as a scavenging receptor for CXCL12, sequestering the chemokine into lysosomal compartments, thereby negatively regulating CXCL12-CXCR4 signaling. Studies in mice demonstrated that CXCR7 expression was upregulated in vascular endothelium during EAE [23]. This was due, in part, to the actions of the T-cell cytokines IL-1*β* and IL-17, which enhanced CXCR7 expression and CXCL12 internalization in BMECs* in vitro*. Administration of the CXCR7 antagonist CCX771 diminished the clinical symptoms of EAE by preserving CXCL12 polarity at the BBB, thereby reducing parenchymal infiltration of autoreactive leukocytes. This preservation of CNS immune privilege was further shown via* in vivo* diffusion tensor imaging to prevent axonal injury during EAE [40].

In addition to direct regulation via CXCR7, CXCL12 is also indirectly regulated at the BBB by mediators of endothelial cell biology. In particular, we recently reported that sphingosine-phosphate-1 receptor 2 (S1PR2) signaling in neurovascular cells contributed to BBB dysregulation during CNS autoimmunity [41]. S1PRs at the BBB respond to the bioactive lipid metabolite S1P, which is expressed broadly by endothelial cells, erythrocytes, and neuronal lineage cells. In BMECs, S1PRs regulate a variety of cytoskeletal processes that influence intercellular junctions, paracellular permeability, and apicobasal polarity. In our study, S1PR2 signaling in CNS vascular endothelium was associated with increased BBB permeability and loss of CXCL12 polarity in mice with EAE and in a human* in vitro* BBB model [27, 41]. Pharmacological blockade or genetic deletion of S1PR2 ameliorated clinical symptoms and parenchymal leukocyte infiltrates and was shown to preserve BBB integrity and CXCL12 polarity in CNS microvessels. S1PR2 dysregulation of endothelial barrier integrity was associated with disassembly of endothelial AJs and was dependent on signaling through the cytoskeletal regulatory GTPases RhoA and CDC42, as well as the caveolin-mediated endocytic pathway. Of note, changes to BBB permeability and adherens junction formation were always associated with loss of CXCL12 polarity, indicating that CXCL12 display on basolateral surfaces of BMECs can be controlled both directly via CXCR7-mediated relocation and by general disruption of AJ-mediated apicobasal polarity via cytoskeletal and endocytic regulatory pathways.

S1PR2 signaling at the BBB may prove to be a particularly attractive therapeutic target, due to its recently discovered contribution to sexually dimorphic patterns of CNS autoimmunity. MS exhibits a strong sexual bias in humans; women comprise the majority of patients and are more susceptible to remitting-relapsing forms of the disease. The EAE model in the SJL mouse strain exhibits similar sexual dimorphism, with female mice exhibiting enhanced susceptibility to EAE and experiencing a remitting-relapsing course of clinical disease. In contrast, males do not develop remitting-relapsing disease and instead exhibit variable disease courses, including complete resistance to EAE or standard monophasic disease progression, depending on such factors as method of EAE induction or age of experimental animals [42–44]. Our recent study found sexually dimorphic expression of S1PR2 in the SJL mouse strain, with increased expression in the cerebella of naïve female SJL and in cerebella and spinal cords of female SJL with EAE compared to males. In addition, we reported enhanced expression of S1PR2 in cerebella of female patients with MS compared to males. Remarkably, the enhanced expression of S1PR2 in EAE-susceptible CNS regions of female SJL mice was associated with similarly sexually dimorphic pathological expression of CXCL12. Naïve female SJL mice exhibited dysregulated CXCL12 polarity in lower CNS regions, contributing to their enhanced susceptibility to EAE, and this dysregulation was further exacerbated compared to males during disease. S1PR2 was found to contribute significantly to sexually dimorphic differences in CXCL12 expression and localization, as pharmacological blockade and genetic deletion of S1PR2 signaling preserved CXCL12 polarity during EAE, resulting in ameliorated disease. Together, these findings suggest that targeting S1PR2 signaling may be a powerful tool in addressing sex-specific disease processes in MS, including the dysregulation of chemokine signals that regulate CNS immune privilege.

## 4. Chemokine-Mediated Repair in the Adult CNS

In MS, myelin destruction and loss of oligodendrocytes lead to motor and sensory disability. A majority of MS patients present with a remitting-relapsing form of the disease, in which periods of inflammation and demyelination are followed by partial recovery [45]. Following neuroinflammation, mechanisms that parallel developmental pathways are activated to facilitate repair. Neural precursors expressing NG2 chondroitin sulfate proteoglycan that give rise to mature oligodendrocytes are found in the ventricular zones of the CNS by embryonic days 12–14. In the final stages of differentiation, oligodendrocyte precursor cells (OPCs) begin to express maturation markers including myelin basic protein (MBP) and myelin oligodendrocyte glycoprotein (MOG) [46]. Similarly, using a model of cuprizone- (CPZ-) mediated demyelination, Patel et al. demonstrated that astrocyte-expressed CXCL12 was necessary for maturation of NG2^+^ OPCs that express CXCR4 during remyelination in adult mice [30, 31]. Following 6 weeks of CPZ exposure, CXCL12 and CXCR4 mRNA were significantly elevated in the corpus callosum (CC), the primary CNS region affected by CPZ intoxication. Additionally, CXCL12^+^GFAP^+^ astrocytes and CXCR4^+^NG2^+^ OPCs were highest in areas with the most extensive demyelination, the caudal CC [30, 47]. Inhibition of CXCR4 signaling, either via pharmacologic antagonism with AMD3100 or via* in vivo* RNA silencing, prevented OPC maturation and remyelination [30], suggesting that CXCL12-CXCR4 signaling is required for the development of mature oligodendrocytes and repair following CNS injury.

During CNS tissue damage, many inflammatory cytokines are rapidly induced and have diverse roles, with potentially detrimental acute consequences but beneficial effects in CNS recovery [48]. While inflammation and repair are two distinct processes, they are both regulated by inflammatory cytokines, like tumor necrosis factor (TNF-α), which influences tissue-infiltrating leukocytes and development of OPCs. Targeting of TNF-α has been successful in treating peripheral autoimmune diseases like rheumatoid arthritis [49], psoriasis [50], and inflammatory bowel disease [51]; however, administration of a recombinant TNF receptor immunoglobulin fusion protein worsened MS exacerbations [52]. Shortly after this study was completed, it was shown that TNF-α signaling through TNFR2 is essential for proliferation and differentiation of OPCs during remyelination in the CPZ model, perhaps explaining the failure of anti-TNF-α strategies for treating MS [53]. Our follow up study revealed that several CNS cells express both TNFR1 and TNFR2 following CPZ-induced demyelination and that TNFR2 signaling, specifically in astrocytes, was critical for CXCL12 expression. Mice deficient in TNFR2 had reduced CXCL12 expression in astrocytes during demyelination, leading to a reduction in CXCR4^+^ OPCs due to a lack of CXCL12-mediated proliferation. In rescue experiments, lentiviral delivery of CXCL12 to the demyelinated CC restored OPC proliferation and remyelination in TNFR2-deficient mice. Further, stereotactic injection of postnatal astrocytes infected with a lentivirus expressing CXCL12 restored MBP expression in the CC following chronic demyelination, suggesting astrocyte CXCL12 is sufficient for myelin production [31]. These data indicate that activated astrocytes express TNFR2, which binds TNF-α to increase the levels of CXCL12 and promote OPC proliferation and differentiation.

**Figure 1:**
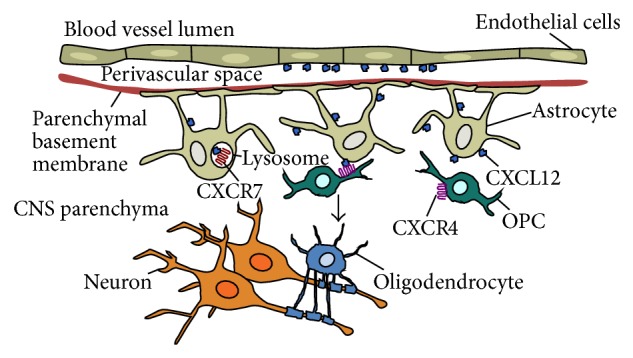
Chemokines mediate repair in the adult CNS. Following demyelination, CXCL12 and its receptors, CXCR4 and CXCR7, are upregulated on astrocytes and endothelial cells. CXCL12 binding to CXCR4 on OPCs induces proliferation and maturation into myelin-producing oligodendrocytes. CXCR7 regulates CXCR4 activation by sequestering CXCL12 into lysosomal compartments for degradation.

While CXCL12 is indispensable for the plasticity of the demyelinated adult CNS, there are several factors that regulate CXCL12 expression during neuroinflammation (Figure 1). In addition to soluble factors, CXCR7, an alternative scavenger receptor, works to sequester and degrade CXCL12 [17, 54], regulating activation of CXCR4. CXCR7 is highly expressed during CPZ-mediated demyelination and expression subsides during remyelination, while levels of CXCR4 and CXCL12 remain elevated. This suggests that downregulation of CXCR7 is necessary for CXCL12-CXCR4 binding during repair. To determine if high levels of CXCR7 might regulate CXCL12 expression during demyelination, Williams et al. administered a small molecule inhibitor of CXCR7, CCX771, versus a vehicle control and found that antagonism of CXCR7 during late CPZ-mediated demyelination resulted in increased expression of CXCL12 and an upregulation of activated CXCR4 in OPCs [32].* In vitro* experiments determined that CCX771-mediated regulation of CXCL12 was due to a decrease in CXCL12 internalization, suggesting that CCX771 works to limit CXCL12 targeting to lysosomal compartments for degradation.* In vivo* CXCR7 antagonism during demyelination led to an increase in OPC proliferation and in mature oligodendrocytes within demyelinated lesions. Further, CCX771 treatment during CPZ-induced demyelination enhanced remyelination, which, through the use of AMD3100, was shown to be CXCR4-dependent suggesting that the CCX771-mediate increase in CXCL12 expression during demyelination has potentially beneficial consequences [32]. These findings are significant as current treatment strategies for MS employ immunosuppressive compounds and do not promote repair. Further, while OPCs are found in MS lesions they are developmentally arrested [55, 56], remyelination gradually fails, and demyelination persists, which leads to progression of clinical disease [57]. Since CXCR7 regulates CXCL12-CXCR4-mediated CNS myelin repair, it may, therefore, serve as a therapeutic target to promote OPC differentiation and remyelination in the adult CNS.

## 5. Chemokines Mediate Neuroprotective T-Cell Recruitment and Activation during West Nile Viral Infections of the CNS

Lymphocyte recruitment into the CNS following viral infections is necessary to control viral replication and, in many cases, prevent death. However, viral infections of the CNS pose unique challenges to the immune system with regards to controlling and eliminating the invading pathogen. First, lymphocyte trafficking to the CNS during inflammation is complicated by the BBB, which prevents most lymphocytes from entering the CNS, and second, the CNS contains terminally differentiated cells that are susceptible to injury caused by the infiltration of pathogen-specific as well as bystander T lymphocytes. Thus, regulatory mechanisms are essential to ensure the appropriate CNS entry of virus-specific T cells and that their presence within the CNS does not induce immunopathology. Chemokines have been recognized as key regulators of leukocyte trafficking from the microvasculature into the CNS and are crucial in coordinating protective immune responses during CNS viral infection [58].

WNV, a neurotropic flavivirus, has emerged globally as a significant cause of viral encephalitis [59]. In the CNS, WNV targets neurons, including cortical, midbrain, cerebellar, and spinal cord neurons, leading to their injury or death [60–62]. The clearance of WNV within the CNS relies heavily on cell-mediated immune responses that promote the migration and effector function of T cells in the CNS parenchyma. Shortly after local viral replication begins, virus-specific CD8^+^ T cells traffic into WNV-infected CNS. Initially, studies suggested that the infiltrating CD8^+^ T cells may have both a neuroprotective and a neuropathologic role due to injury of WNV-infected neurons [63]; however, studies using targeted deletion of T-cell chemoattractants to inhibit leukocyte trafficking indicate that the CNS entry of virus-specific CD8^+^ T cells is essential for clearance of WNV within the CNS.

Proinflammatory chemokine expression is strongly induced within the CNS of WNV-infected mice and coincides with the infiltration of mononuclear leukocytes [28, 64]. The chemokines CCL3-5 bind to the chemokine receptor CCR5, which is upregulated within the CNS after WNV infection. The deletion of CCR5, which is expressed on activated T cells and macrophages, decreases efficient leukocyte trafficking and viral clearance within the CNS during WNV infection [64]. CXCL10 expression is also induced following WNV infection by virally infected neurons [28]. Cerebellar neurons express higher levels of CXCL10 compared with cortical neurons, which results in enhanced trafficking of CXCR3-expressing WNV-specific T cells into the hindbrain versus the forebrain [29]. During EAE, neuronal precursor cells also express CXCL10 within the subventricular zone (SvZ) which results in the preferential localization of activated T cells [65] confirming the potent ability of neuronal cells to direct inflammatory cell infiltration. However, this differential pattern in CXCL10 expression during WNV infection may be due to differences in innate neuronal expression of viral sensing proteins [66]. The loss of CXCL10 or CXCR3 via targeted deletion or antibody administration results in decreased recruitment of WNV-specific CD8^+^ T cells in the CNS, especially within the cerebellum, increased viral loads, and enhanced mortality [28, 29], establishing that WNV-infected neurons directly induce the recruitment of virus-specific T cells in a region-specific manner for the purpose of viral clearance.

CXCL10 is also involved in neuronal apoptosis pathways via activation of CXCR3 by neurons. Previous studies demonstrated that treatment of CXCR3-expressing neurons with CXCL10 results in a caspase-3 dependent apoptotic cell death [67]. Therefore, the chemokine required for the recruitment of effector immune cells by virally infected neurons for their survival might also promote their death. Thus, virally infected neurons, in addition to CXCL10, also express TNF-α, which down-regulates CXCR3 expression in both infected and uninfected neurons [68]. TNF-α-mediated loss of CXCR3 interferes with caspase-3 activation and apoptotic death [68]. Thus, neurons can both facilitate an appropriate antiviral inflammatory response and prevent injury from the proapoptotic effects of the required inflammatory chemokine.

Constitutive chemokines, which are normally expressed by secondary lymphoid tissues as well as the CNS, function in regulating immune surveillance and primary immune responses. As described above, CXCL12, which is expressed by endothelial cells of the CNS microvasculature, regulates the trafficking of leukocytes into the CNS parenchyma during neuroinflammatory diseases [24–26, 37, 40]. Infiltrating lymphocytes cross endothelial barriers within the leptomeninges and enter the CNS via crawling along abluminal surfaces, generating the dense perivascular infiltrates typically observed during neuroinflammatory diseases [69–71]. CXCL12 expression at the microvasculature localizes immune cells, promoting their interaction within perivascular spaces, regulating the entry of fully activated WNV-specific T lymphocytes during WNV encephalitis [25, 26].

Within lymph node tissues, CXCL12 expression mediates the homing and localization of mononuclear cells to lymphoid compartments [72, 73] from which effector lymphocytes are released via changes in CXCR4 expression, the receptor for CXCL12 [74–76]. Similar to its role within the periphery, CXCL12 expression within the CNS functions to retain leukocytes that have migrated into the perivascular spaces of the CNS microvasculature. During WNV encephalitis, levels of abluminal CXCL12 along the microvasculature drop when compared with uninfected counterparts suggesting a mechanism for promoting leukocyte entry for viral clearance [26]. Consistent with this, the blockade of CXCL12 signaling with a CXCR4 antagonist promotes leukocyte entry into the CNS parenchyma and results in improved viral clearance, decreased immunopathology, and enhanced survival during WNV infection [26]. These data provide evidence that the parenchymal location of virus-specific T cells is essential to effectively clear virus and reduce pathology. In addition, they suggest that contrary to the role of CXCL12 within secondary lymphoid tissues, the movement of leukocytes into and out of CNS perivascular spaces relies on regulation of CXCL12 expression rather than CXCR4, which may have an important role in antigen recognition within the CNS.

How CXCL12 expression is regulated is not completely understood. Several studies have demonstrated that members of the TNF superfamily, including TNFα/TNFR and CD40/CD40L, interact and upregulate CXCL12 in various cell types [77, 78]. CD40 is a cell surface receptor that is expressed by numerous immune cells, including dendritic cells, B cells, and macrophages as well as endothelial cells [79]. Ligand binding of CD40 within CNS microvasculature is associated with the retention of myelin-specific T cells in EAE [80, 81]. During WNV infection, the targeted deletion of either CD40 or TNFR-1 decreases CD8^+^ T-cell trafficking into the CNS parenchyma [82, 83]. These studies suggest that TNF ligand/receptor superfamily interactions facilitate T-cell migration across the BBB to control WNV infection; however, further studies are needed to determine whether these molecules exert their T-cell trafficking effects via regulation of CXL12 expression.

**Figure 2:**
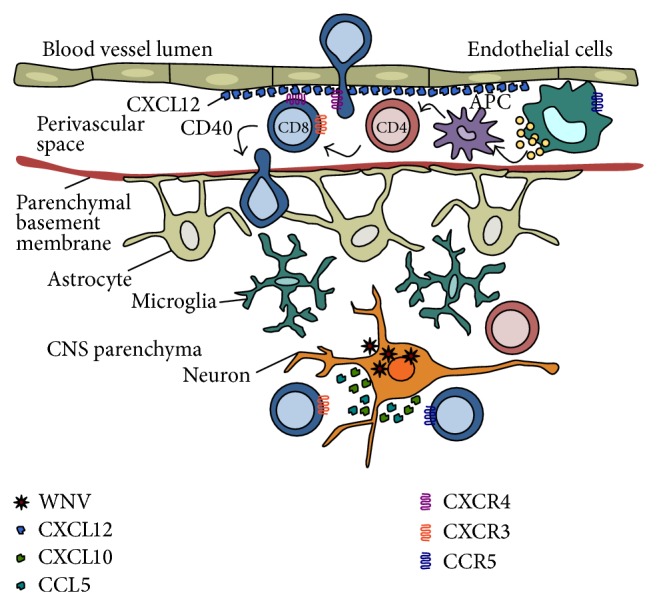
Neuroprotective roles of chemokines in response to viral infection of the CNS. Functional roles of chemokines in attracting and activating lymphocytes into the CNS during acute viral infection. Monocytes are attracted into the CNS via the chemokine CCL5 and its receptor CCR5. Macrophages produce IL-1*β* within the CNS and ensure full activation and CXCL12-mediated interactions of infiltrating leukocytes. CXCR4-expressing lymphocytes are captured by endothelial expression of CXCL12 within the perivascular spaces, which promotes CD4^+^ T-cell help to infiltrate CD8^+^ T lymphocytes. In addition to full activation, lymphocyte egress from perivascular spaces requires CD40. During the acute stage of disease, virally infected neurons secrete CXCL10 and CCL5 that attract activated T lymphocytes bearing the receptor CXCR3 and/or CCR5 into the parenchyma. CD8^+^ and CD4^+^ T lymphocytes mediate viral control through direct cytolytic activity and/or cytokine secretion.

CXCL12-mediated retention of leukocytes within the perivascular compartment promotes leukocyte interactions that ensure the full activation of virus-specific lymphocytes and improves their ability to migrate out of the perivascular space into the CNS parenchyma (Figure 2). CD4^+^ T cells assist the full activation, migration, and positioning of virus-specific CD8^+^ T cells within the CNS, which are essential to effectively clear virus [84–86]. In the absence of IL-1 signaling, antigen presenting cells fail to be fully activated, which is required for CD4^+^ T-cell help to restimulate infiltrating, virus-specific CD8^+^ T cells [84, 87]. In addition to inefficient viral clearance, the loss of IL-1 signaling results in increased leukocyte entry into the CNS parenchyma, including bystander, nonspecific T cells, increased immunopathology, and enhanced mortality during WNV infection [84, 88]. Proinflammatory chemokines including CCL2, CCL5, and CXCL10 were significantly upregulated following infection yet were ineffective in recruiting leukocytes that mediated viral clearance. Infiltrating macrophages produce IL-1 within the CNS promoting increased expression of CXCL12 along the microvasculature. This in turn enables leukocyte accumulation to ensure the selective entry of fully activated virus-specific T cells into the parenchyma, which imparts protective CNS inflammation during WNV infection.

Within the CNS there are multiple regulatory mechanisms that govern leukocyte trafficking from the microvasculature into the CNS parenchyma. Chemokines are critical coordinators of these particular immune responses during CNS infection by neurotropic viruses. Taken together, these molecular and cellular events are exceedingly efficient in clearing viral pathogen while limiting CNS immunopathology.

## 6. Concluding Statements

The hallmark of neuroinflammation is an influx of leukocytes through the BBB. Depending on the disease, the presence of leukocytes can have beneficial or detrimental effects on disease outcome. Chemokines have a protective role within the CNS through their ability to orchestrate leukocyte entry and interactions at the endothelial barriers of the CNS and initiate CNS repair of damaged tissue within the CNS parenchyma. CXCL12 expression along the CNS microvasculature leads to neuroprotection by limiting the parenchymal infiltration of autoreactive leukocytes and ensuring successful interactions for the release of fully activated virus-specific leukocytes necessary for swift viral clearance. Following neuroinflammation, CXCL12 expression, within the white matter of the CNS parenchyma, is critical in promoting OPC maturation and remyelination. The ability of CXCL12, and other proinflammatory chemokines, to mediate leukocyte influx and repair from inflammation mediated tissue damage suggests that chemokines act as an interpreter of an immune reaction and transform the information in neuroprotective mechanisms during infection and disease. However, the protective and immunomodulatory role of chemokines within the CNS during neuroinflammation is still not fully elucidated. For instance, in neurodegenerative diseases such as Alzheimer's disease, recent reports indicate that chemokines accelerate amyloidosis via regulation of resident microglia [89]. Moreover, chemokines may orchestrate intercellular interactions within the CNS, specifically T lymphocyte and APC interactions, which may achieve immune skewing at the microvasculature or other CNS barriers. These chemokine-mediated leukocyte interactions may promote the activation or expansion of functionally precommitted immune cells with the desired phenotype or the suppression of cells with an inappropriate phenotype at the border of the CNS. Additionally, chemokine receptor signaling may modulate the downstream expression of appropriate growth factors necessary for repair within the parenchyma following inflammation. Finally, the protective and immunomodulatory role of chemokines within the CNS may extend beyond this immune-privileged site and be relevant for the immune regulation within other systems such as the eye, intestine, lung, tumor, and chronic infection.
